# Potential protective properties of flax lignan secoisolariciresinol diglucoside

**DOI:** 10.1186/s12937-015-0059-3

**Published:** 2015-07-28

**Authors:** Muhammad Imran, Nazir Ahmad, Faqir Muhammad Anjum, Muhammad Kamran Khan, Zarina Mushtaq, Muhammad Nadeem, Shahzad Hussain

**Affiliations:** 1 Institute of Home and Food Sciences, Faculty of Science and Technology, Government College University, Faisalabad, 38000 Pakistan; 2 Department of Dairy Technology, University of Veterinary and Animal Sciences, Lahore, Pakistan; 3 Department of Food Science and Nutrition, College of Food and Agricultural Sciences, King Saud University, Riyadh, Saudi Arabia

**Keywords:** Flaxseed, Processing, Lignan, Precursors, Diet, Therapy, Maladies

## Abstract

Lignans are a group of phytonutrients which are widely distributed in the plant kingdom. Flaxseed is the richest source of providing lignan precursor such as secoisolariciresinol diglucoside (SDG). This article reviews the studies relevant to experimental models in animals and humans demonstrating the possible nutraceutical actions of SDG to prevent and alleviate lifestyle-related diseases. A local and international web-based literature review for this project was carried out to provide information relating to the study. The major key word “*SDG*” was selected to gather information using the electronic databases pertaining to the current state of flaxseed lignans composition, bioactive compounds, metabolism and to find out their role in terms of chemopreventive action. The extraction methods vary from simple to complex depending on separation, fractionation, identification and detection of the analytes. The majority of studies demonstrate that SDG interferes with the development of different types of diseases like cardiovascular, diabetic, lupus nephritis, bone, kidney, menopause, reproduction, mental stress, immunity, atherosclerosis, hemopoietic, liver necrosis and urinary disorders due to its various biological properties including anti-inflammatory, antioxidant, antimutagenic, antimicrobial, antiobesity, antihypolipidemic and neuroprotective effects. Moreover, SDG has a defending mediator against various cancers by modulating multiple cell signaling pathways. As discussed in this review, SDG has shown therapeutic potential against a number of human diseases and can be recommended for discerning consumers.

## Background

The flaxseed (*Linum usitatissimum* L.) is the seed from the flax plant, an annual herb which belongs to *Linaceae* family with more than 200 species. The Latin name of flaxseed means “very useful”, and it has brown and golden varieties. The shape of flaxseed is flat or oval up to 4–6 mm size with a pointed tip. Flaxseed has been a part of human diet for thousands of years in Asia, Europe, Africa, North America and more recently in Australia. The world flaxseed production remained static about 2.6 million tonnes as compared with other oilseed crops and represents 1 % of total world oilseeds supply. Currently, flaxseed has been the focus of increased interest in the field of diet and disease research due to the potential health benefits associated with some of its biologically active components such as dietary fiber (25–28 %) and α-linolenic acid (50–55 % of total fatty acids composition) [1].

Among the compounds that present biological activity, phenolic compounds are of special interest. Lignans, very complex classes of bioactive polyphenolic phytochemicals, formed by the coupling of two coniferyl alcohol residues are widely distributed in the plant kingdom [2]. There are two general types of lignans: i) those found in plant seeds like secoisolariciresinol diglucoside (SDG), isolariciresinol, matairesinol, lariciresinol and ii) those found in animals and humans known as mammalian lignans [3]. Phenolic lignans are found in most fiber-rich plants, including pumpkin seed, sesame seed, grains such as wheat, barley, rye and oats; legumes such as beans, lentils, and soybeans; and vegetables such as garlic, asparagus, broccoli, and carrots. Flaxseed is particularly the richest known source of lignans (9–30 mg per g), with lignan production at 75–800 times that of other oil seeds, cereals, legumes, and fruit and vegetables [4]. The principal dietary lignan present in flaxseed is SDG which occurs as a component of a linear ester-linked complex. Chemically, the C_6_-OH of the glucose of SDG is esterified to the carboxylic acid of hydroxymethylglutaric acid. Accumulation of SDG is coherent with LuPLR gene expression and synthesis of PLR enzyme during mature seed development [5]. The understanding of the action mechanism of these SDG compounds is crucial for their possible exploitation as neutraceutical supplement in biological system.

## Methods for literature search

The search for relevant literature was conducted using electronic databases which were searched for peer-reviewed journal articles and further expanded our search to latest available books of food and nutrition by visiting websites and by consulting with reference librarians and experts in the field. Specifically, various words such as ‘Flaxseed’, ‘Processing’, ‘Lignan’, ‘Precursors’, ‘Diet’, ‘Therapy’, ‘Maladies’ were searched in keyword, title, or abstract of an article or related books. Particular databases used were: ‘American Society of Agriculture and Biological Engineers (ASABE)’, ‘SAGE online journals’, ‘Nature Publishing’, ‘Cambridge University Press’, ‘Elsevier (Science Direct)’, ‘Jastor’, ‘Science Online’, ‘Springerlink’, ‘Taylor and Francis Journals’, ‘Wiley-Blackwell Journals’, ‘Beech Tree Publishing’, ‘ISI Web of Knowledge’, ‘Agricola’, ‘Agris’, ‘Biomed Central’, ‘Cancer.gov’, ‘Directory of Open Access Journals’, ‘Google Directory’, ‘High Wire Press’, ‘Pubmed’, ‘SciELO-Scientific Electronic Library Online’, ‘Scopus’ and ‘Health Source’. Reference lists of all relevant articles were examined for additional studies. After collection of search literature, abstracts and articles were classified as highly relevant, moderately relevant or irrelevant. Highly relevant articles were those in which direct extraction of SDG and derived mammalian lignans from flaxseed or flaxseed by-products was carried out. Moderately relevant articles were those that failed to meet the ‘highly relevant’ criteria yet provided background information regarding other phenolic compounds of flaxseed. Inclusion criteria were based on animal and human models with respect to SDG treatment for different maladies. All other articles were deemed irrelevant to this review and considered as exclusion criteria based on insufficient information was available to permit methodological evaluation for SDG extraction, analysis and processing or if complex multimodal efficacy programmes were used. Three subject experts from the institute were invited for independently applied the inclusion/exclusion criteria to papers identified from the literature search as highly relevant before combining results. A consensus method was used to solve any dispute regarding the inclusion or exclusion of a study. A fourth reviewer was consulted to resolve disagreements [6]. On the basis of the criteria specified above, as of May of 2015, total 220 while 140 moderate relevant articles were found in electronic database. From which only 70 highly relevant articles were part of results.

## Extraction, isolation and purification of SDG

Most of the analytical techniques for the extraction, isolation, and purification of SDG have been conducted on whole flaxseed and defatted flaxseed meal. Flaxseed lignans especially SDG occur in ester-linkage form in the hulls enclosing the seeds, which require special pretreatments including various steps and combinations of enzymatic, acidic, and alkaline hydrolysis before extraction and analysis [7, 8]. Bakke and Klosterman firstly reported a laboratory process for extracting SDG from defatted flaxseed meal using equal parts of 95 % ethanol and 1,4-dioxane [9]. Furthermore, several scientists used a variety of organic solvents mixture such as of methanol, acetone, isopropanol and butanol to extract SDG [10]. Base hydrolysis treatments include sodium, calcium, ammonium and potassium hydroxides for the liberation of SDG from flaxseed lignan polymer [7]. Recent studies have evidenced that the applications of novel technologies for extraction of SDG compounds from flaxseed oilcake is of particular interest within the context of green chemistry as these technologies use reduced solvent consumption, reduced extraction time, lower temperature, less thermal damages to the extract and minimize the loss of bioactive compounds in comparison to other conventional published methods [11]. Considering the importance of the phenolic fraction of flaxseed, high performance analytical methods have been developed to characterize its complex lignan polymer pattern. The analytical methods depending upon separation methodology for SDG can be categorized into chromatographic and non-chromatographic techniques. In most research studies, gas chromatography, high-performance liquid chromatography coupled with photodiode array detector and mass spectrometric procedures have been used for the quantification and analysis of SDG purity [12, 13].

### SDG-enrich foods

SDG can be successfully supplemented in numerous foods due to high stability percentage in finish products. SDG has been found to withstand baking temperatures (250 °C) and can be incorporated in cereal-based bakery products [14]. The SDG concentration of doughs, baked rye breads, graham buns, and muffins was found relatively stable during storage at room temperature for 1 week and at −25 °C for 2 months, respectively [15]. Similarly, macaroni fortified with whole ground flaxseed at levels of 10 to 20 %, dried under ultrahigh temperature (90 °C) and stored for 32 weeks under ambient conditions possessed approximately 80 to 95 % of the SDG contents [16]. Quantitative recovery of SDG from the commercially prepared breads was observed when product formulation was supplemented with pure SDG. However, 73–75 % recovery of SDG was noted from baked bread samples which contained flax meal or aqueous alcohol extracts in product recipe. The extent of grinding of the flaxseed was also shown to have a significant effect on the recovery of SDG from both flax meal breads and baked goods, with extraction of SDG from finely ground samples greater than that from course material [17]. A great part of the SDG content (89 %) was found stable during the heat treatment of ground flaxseed, either alone or as an ingredient in bread, under various conditions of temperature and during storage for several days [18]. Added SDG was retained in the whey fraction and 6 % was found in the cheese curd while up to 25 % of added SDG was lost in whey-based drinks during storage of 6 months at 8 °C [19]. SDG shows natural antioxidant mechanism in foods and prevents oxidation reactions resulting in enhanced shelf life of foods. The ethanolic flaxseed extracts enriched with SDG compounds exhibit antioxidant activity during frozen storage of meat products. However, antioxidant efficiency of the SDG-enriched extracts seems to depend on chemical composition of raw material and flax variety [20]. Moreover, thermal processing has been responsible for slight increase in extractability level of the lignans from raw flaxseed meal which is may be due to increasing porosity of the heated seeds. One recent scientific study reported that the flaxseed samples heated at 250 °C for 3.5 min possessed high SDG contents (1200 mg/100 g) when compared from unheated flaxseed samples (1099 mg/100 g) [14]. However, to conserve the relatively high content of lignans during production of commercial foods products, the initial raw material composition, the water content and the applied temperatures have to be considered.

### Bio-activation of SDG

The biological activity of SDG results from their conversion to the mammalian lignans enterolactone (EL) and enterodiol (ED) by the intestinal microflora in the upper part of the large bowel. The mammalian lignans differ from plant lignans in that mammalian lignans have the hydroxyl groups in the meta position while plant lignans have the oxygenated substituents primarily in the para positions [21]. The mammalian lignans, firstly identified in humans and animals in 1980 [22], are formed in the human body by the action of diverse phylogenetically bacterial strains dominating Peptostreptococcus sp. SDG-1 and Eubacterium sp. SDG-2 present in the gastrointestinal (GI) tract through hydrolyzing the sugar moiety of plant lignan precursors followed by dehydroxylation and demethylation process [23]. Many factors, in addition to diet, such as intestinal microflora, smoking, antibiotics, and obesity affect circulating lignan levels in the body [24]. Due to variations in these factors, large differences among individuals have been observed in lignan bio-activation in urine, fecal and blood samples. Different studies have analyzed human intake of lignans. However, so far research has not shed light on what proportion of ingested mg of plant lignans is metabolized in the gut, absorbed and finally reaches target tissue [23]. Mammalian lignan production from intake of whole or milled flaxseed supplemented baked products is dependent on time and dose but not on processing. The processed flaxseed supplemented muffin or bread did not affect the quantity of lignan excretion in womens which reflect stability and bioavailability of plant and mammalian lignans in human biological metabolism [25].

## Chemopreventive properties of SDG

Flaxseed Lignan precursors and their mammalian metabolites may be appreciated as health promoting dietary micronutrients having chemopreventive properties in animals and humans by utilizing as nutraceutical agent against different chronic diseases like cancer, atherosclerosis, diabetes, kidney disorders and lupus nephritis [26]. Some of the health effects of flax SDG are discussed in the following sections.

### Anti-cancer effect

The animal and human studies have shown the prevention role of SDG against some cancers (breast, lung and colon) as a result of its strong anti-proliferative, antioxidant, anti-oestrogenic and/or anti-angiogenic activity. It is proposed that the anticancer activity of SDG is associated with the inhibition of enzymes involved in carcinogenesis. The growth of crypts and crypts foci are the earlier risk factor for colon cancer. The animal studies have showed that flaxseed SDG and lignan supplementation in rat’s diet resulted in aberrant crypts and it foci showing the anticancer role of these molecules [27]. Similar, the anti carcinogenic effect of SDG molecule has been observed in pulmonary metastasis, mammary gland and breast cancer metastasis. The studies showed that the supplementation of SDG in mice diet resulted in reduction of volume, area and numbers of tumors significantly as compared to control mice group. The two week supplementation of SDG in mice diet led to 22 % more pulmonary metastasis tumors in melanoma cells than average tumors as compared to control group having 59 % more tumors than average [28]. The SDG of flaxseed are proven to initiate the differentiation of enhancement of terminal end buds in mammary gland and thus have role in prevention of breast cancer. The series of studies have shown that progression of N-methyl-N-nitrosourea-induced mammary tumorigenesis results in development of carcinogenesis and SDG has proven to delay the progression of this phenomenon by regulating the terminal end bud differentiation [29, 30]. There are several possibilities that how SDG can biologically involve in delay or prevention of carcinogenic phenomenon. It is supposed that plasma insulin-like growth factor I, endothelial growth factor is responsible for risk factor of breast cancer progression and SDG can lower these growth factors [31, 32]. Another possibility of prevention role of SDG is in mediation of Zn concentration which observed more in breast cancer tissue compared to tissue of normal breast which may provide protection against breast cancer by limiting angiogenesis in such cases [33].

Human studied have shown that there could be consisted possibility of correlation between SDG and cancer. SDG may affect hormonal levels and may influence cancer progression. The potential effects of SDG are attributed to concomitant fat restriction. The research studies conducted by Demark-Wahnefried and co-workers [34, 35] on prostate cancer proliferation in human subjects have shown that flaxseed-supplemented could affect the biomarkers of prostatic neoplasia. A randomized controlled trial test on 161 prostate cancer patients for 30 days before prostatectomy were conducted by assigning diet without flaxseed supplementation and flaxseed-supplemented of 30 g/day. The proliferation (Ki-67, the primary endpoint) and apoptosis were assessed for tumor development. Proliferation rates were significantly lowers among men assigned to the flaxseed supplemented diets. Their findings suggest that flaxseed is associated with biological alterations that may have prevention role against prostate cancer. The studies from two research groups of Boccardo et al. [36] and Pietinen et al. [37] have demonstrated a strong correlation between SDG metabolites and breast cancer in women. The serum level of intestinal microbial derived SDG metabolites enterodial and enterolactone have inversed association with breast cancer when studies were conducted on 508 breast cancer women cases. The overall animal and human studies have been suggested SDG and its metabolites may provide prevention against cancer as result of its antioxidant activity or ability to inhibit enzyme action involved in steroid hormone metabolism.

### SDG and heart diseases

The major hearts diseases are stock, coronary artery disease, peripheral artery disease which are resulted from oxidative stress, inflammation, obesity, diabetes, dyslipidemia and hypertension and contribute to an atherogenic environment that promotes the development of myocardial infarction and stroke, leading causes of mortality among industrialized nations [38, 39]. The animal and human studies have suggested that SDG and its metabolites mediate the serum total cholesterol, low density lipoprotein, total cholesterol and high density lipoprotein ratio which lead to less androgenic complication and antioxidative prevention [40]. A series of research studies indicates that regular flaxseed with α-linolenic acid and flax lignan polymer (containing 34–38 % SDG, 10–11 % 3-hydroxy-3-methylglutaric acid and 15–21 % cinnamic acids) as potential bioactive components or purified SDG in equimolar concentration have similar antiatherogenic effects [41].

Similarly, the human studies showed the SDG as potential cardiovascular protector by mediating the mechanisms of total cholesterol, LDL-cholesterol, HDL-cholesterol, triacylglycerides and glucose metabolism. It was observed that 20 hypercholesterolaemia and hypertriglyceridaemia subjects receiving 600 mg SDG per day for 8 weeks led to significant reductions in total cholesterol, LDL-cholesterol and glucose concentrations compared with the placebo group [42]. Several other studies have shown that SDG anti-cardiovascular effects are associated with enterolactone mediating increased expression of vascular endothelial growth factor, endothelial NO synthase and haeme oxygenase-1 mediated myocardial angiogenesis. Overall, the majority of studies that used purified SDG found improvements in markers of CVD [43].

### Anti-diabetic action of SDG

Diabetes is a metabolic syndrome and is characterized by increases in central adiposity, serum tirglycerides, serum glucose, blood pressure, inflammation and decreases in HDL-cholesterol that elevates risk of insulin resistance [44]. The animal and human studies revealed that high fat diet containing 0 · 5 to 1 · 0 % SDG reduces liver triglycerides content, serum triglycerides, total cholesterol, and insulin and leptin concentrations that resulted in significantly reduced visceral fat gain as compared to group of mice receiving high fat diet without SDG [45]. Another study have shown that female rats receiving glucosuria induced diet with SDG have 80 % less chances of glucosuria as compared to rats have 100 % chances of glucosuria receiving diet without SDG [46]. SDG reduces C-reactive protein concentrations which are associated with insulin resistance and diabetes mellitus in type 2 diabetics [47]. Daily consumption of low-fat muffin enriched with SDG (500 mg/day) for 6 week can reduce CRP concentrations [48]. The earlier studies indicate that flaxseed lignan supplements have beneficial associations with C-reactive protein and also suggest that lignans have possible lipid- and blood pressure-lowering associations [49].

### SDG effect on liver necrosis

The free radicals and reactive oxygen species (ROS) are produced as a result of exogenous chemicals and/or to the endogenous metabolic processes involving redox enzymes and bioenergetic electron transfer in the biological system. These free radicals and ROS thus induce oxidative stress leading to damage of proteins, lipids and nucleic acid and results in cancer, diabetes, atherosclerosis, hepatic diseases. The actions of these molecules can be nullified through antioxidants mechanism [50, 51]. Antioxidant potential of SDG and its metabolites have been reported in several animal and human models. Animal studies have shown that SDG containing flaxseed extract significantly increases the levels of serum ALP, ALT, AST, Bilirubin, blood urea and creatinine with decrease in the levels of total protein and albumin in experimental rabbits exposed with induced hepatotocxicity compared to the control group. The SDG polymer complex can significantly prevent liver and renal damage from paracetamol induced hepatonephrotoxicity in rabbits. Thus, the flaxseed lignan act as a therapeutically useful hepato-nephroprotective agent [52]. Flaxseed supplementation may provide a new therapeutic strategy to reduce hypertriglyceridemia and fatty liver in rats [53]. Another study reported the rabbits exposure to SDG lignans for consecutive 8 weeks to assess histopathologic evaluation score of non-alcoholic fatty liver disease and suggested that SDG (8 mg/kg) can protective from liver diseases [54].

Intervention studies have shown that Flaxseed lignan can decreases liver disease risk factors in moderately hypercholesterolemia mens. The oral administration of SDG would decrease the level of blood cholesterol and liver disease risk factors induced by hypercholesterolemia in humans. Thirty men received placebo and capsules of SDG for 12 weeks and subjects received 100 mg of SDG exhibited a significant reduction in the ratio of low-density lipoprotein/high-density lipoprotein cholesterol, a significant percentage decrease in the levels of glutamic pyruvic transaminase and γ–glutamyl transpeptidase and a significant percentage decrease in the level of γ–glutamyl transpeptidase which may reduce the hepatic diseases risks [55].

### SDG effect on lupus nephritis, bone strength and kidney disease

Faxseed SDG has a therapeutic role in animal and human lupus nephritis. SDG significant delays the onset of proteinuria with preservation in GFR and renal size in a dose-dependent fashion [56]. Purified SDG during early life of a young rat animal sensitize bone strength due to low endogenous levels of sex hormones but having no negative effects on bone strength and bone health, as measures of bone mass in adulthood [57, 58]. SDG in addition with low-dose estrogen therapy, provides the greatest protection against ovariectomy-induced bone loss [59]. However, SDG shows no effect on bone mineral density content, body composition, lipoproteins, glucose level and inflammation [44]. SDG have a beneficial role in chronic renal disease like reduces weight, renal inflammation and lipid peroxides in polycystic kidney disease [60].

### Menopause, urinary composition and reproduction effect

The occurrence of menopause is associated with an increased risk of cardiovascular events and this has partially been attributed to the decline in circulating levels of estrogen. SDG supplementation produces a dose-related cessation or lengthening (by 18–39 %) of estrous cycles, reduces immature ovarian relative weight and delays puberty in experimental animals [61, 62]. The daily consumption of a low-fat muffin enriched with SDG (500 mg/day) for 6 week had no effect on endothelial functioning in healthy postmenopausal women [63]. Dietary flaxseed SDG (600 mg/day) can appreciably improve lower urinary tract symptoms in benign prostatic hyperplasia subjects [64]. Urinary composition or blood levels of radioactive lignans were not affected by the duration of SDG exposure while chronic SDG exposure alters lignan disposition in rats, however; it does not change the metabolite profile [65]. There were no significant effects of exposing male or female offspring to SDG during suckling on any measured reproductive indices [66]. SDG affects the reproductive development of offspring with caution when consuming flaxseed during pregnancy and lactation [67].

### Mental stress and immunity effect

Flax lignan SDG may be associated with the least increase in peripheral resistance as result the greatest reduction in plasma cortisol and the smallest increase in plasma fibrinogen measured during mental stress [68, 69]. SDG has long acting hypotensive effect mediated through the guanylate cyclase enzyme. SDG supplementation shows no significant effects on lymphocyte proliferation indicating that SDG has no side effects on the immune system [70].

## Conclusions

The clinical and laboratory evidences indicate that flaxseed lignans particularly SDG have numerous biological properties that make them unique and very useful in promoting health and combating various diseases. Future biological and interventional studies need the confirmation of SDG as safe and effective for the prevention of lipid, protein and DNA oxidation associated with oxidative stress. The potential health benefits of SDG with supporting evidences from human and animal studies offers suggestions for future research.
